# Assessment of Biological Toxicity and Ecological Safety for Urban Black-Odor River Remediation

**DOI:** 10.3390/ijerph17031025

**Published:** 2020-02-06

**Authors:** Rou-Rou Xu, Zhou-Tao Pei, Wen-Qian Wang, Meng Zhang, Li-Ling Zhang, Jing Zhang, Wen-Qiang Wang, Li-Wei Sun, Yi-Min Zhang

**Affiliations:** 1 School of Energy & Environment, Southeast University, Nanjing 210096, China; 220170522@seu.edu.cn (R.-R.X.); 220170623@seu.edu.cn (Z.-T.P.); 220160595@seu.edu.cn (W.-Q.W.); 220180612@seu.edu.cn (M.Z.); 220180617@seu.edu.cn (L.-L.Z.); 213151155@seu.edu.cn (J.Z.); 213152621@seu.edu.cn (W.-Q.W.); 2 Taihu Lake Water Environment Engineering Research Center (Wuxi), Southeast University, Wuxi 214061, China; 3 Research Center of Watershed Ecological Conservation and Water Pollution Control, Nanjing Institute of Environmental Sciences, Ministry of Ecology and Environment of the People’s Republic of China, Nanjing 210042, China

**Keywords:** urban black-odor river, overlying water, biological toxicity, mutagenicity

## Abstract

The judgment and assessment of remediation effect on urban black-odor river still depend on the physical-chemical parameters and lack in ecological safety effects. A set of combined biological toxicity tests were applied to evaluate the ecological effects of one urban black-odor river before and after the remediation. The special growth rate of *Chlorella vulgaris* and mortality rate of *Daphnia magna* were used to assess acute toxicity. The *Salmonella Typhimurium*/Reverse Mutation Assay was applied to test the mutagenicity. The tests by *C. vulgaris* growth showed that there was no inhibition before and after remediation by overlying water, in contrast promoted the growth of *C. vulgaris*. The tests by *D. magna* showed slight toxicity on site 3# before remediation and nontoxic after remediation. The mutagenicity of organic extracts from overlying water at all sampling sites were positive before remediation, but were eliminated after remediation except from 3 of 4 sites on TA98 strain. The addition of the liver microsomal S9 induced the positive mutagenicity on site 4# compared to S9 absence. The results clarified the applicable and the importance of the biological toxicity tests on assessing the remediation effect and potential ecological risk of urban black-odor river.

## 1. Introduction

Urban water refers to waters within the city that are closely related to urban functions, including rivers flowing through cities, river ditches, lakes, and other landscape water, which is an important part of urban ecosystems. Black-odor river is mainly caused by excessive discharge of pollutants by humans into water and the metabolism of algae and bacteria in waterbody [1].

At present, there is no definite assessment method or evaluation standard for black-odor river. The assessment methods in China are mainly divided into three categories: single index method, comprehensive index method, and other assessment methods.

Single indexes included physical indexes and chemical indexes. Physical indexes are mainly based on vision and smell to judge the degree of black-odor river. Generally, dissolved oxygen (DO) is taken as the main index to judge whether a river is black and odorous [2]. There are other basic water quality parameters for chemical indexes, such as chemical oxygen demand (COD_Cr_), biochemical oxygen demand (BOD_5_), total nitrogen (TN), total phosphorus (TP), ammonia nitrogen (NH_4_^+^-N), and redox potential (ORP).

The commonly used comprehensive indexes are black odor single factor index model [3], organic pollutant index model, and black odor multi-factor weighted index model [4]. In addition to the two assessment methods mentioned above, other model methods such as multivariate nonlinear regression model [5] and remote sensing monitoring and screening method [6] have also been gradually applied to the assessment of black-odor river. In fact, the comprehensive index methods and other assessment methods are based on the establishment of mathematical models, while the data for establishing these mathematical models are still physical and chemical indexes.

Due to the complex types of pollutants contained in black-odor river, including not only toxic and harmful inorganic substances, nitrogen and phosphorus, but also a wide variety of persistent organic pollutants that are difficult to degrade. If only sensory physical indexes and water quality chemical parameters are used to evaluate the remediation effect of black-odor river, the result is obviously lack of ecological safety judgment and do not know whether the remediation process will actually reduce the toxic effects of black-odor river on aquatic organisms. Therefore, there is an urgent need for biological methods that can assess the toxicity and ecological safety of urban black-odor river before and after remediation.

For the evaluation of water toxicity, biological toxicity tests have been proved to be valuable tools for detecting the total biological potential risk of a mixture of pollutants [7]. The Whole Effluent Toxicity Test (WET) proposed by the United States Environment Protection Agency (USEPA) recommends using multiple test species [8], the standardized plants from fresh water and invertebrates, to evaluate the comprehensive toxicity of effluents. Fang et al. [9] found that green algae and crustacean was sensitive to the effluents from electronic and electroplate factories, and they also found that organic pollutants were the main contributing factor to the toxicity of effluents from these factories.

At present, few studies have applied biological toxicity to the evaluation of black-odor river. It is speculated that the biological toxicity tests could be used to evaluate the ecological safety and remediation effect for those black-odor rivers. The present study supposed the water from the black-odor river to be a whole effluent, then their toxic effects to the aquatic system were evaluated, and the reduce of the toxic effects after the remediation were also accessed.

*Salmonella Typhimurium*/Reverse Mutation Assay (Ames test) was designed to detect chemically induced mutagenesis. It can specially detect the mutagenicity of mixed pollutants quickly and accurately [10,11]. So far, it has been widely used to evaluate the mutagenicity of complex mixtures in drinking waters and polluted rivers. Park et al. [12] found that the organic extracts from the drinking tap water in Seoul, Taejon, and Suwon were mutagenic on TA98 without S9 mix and on TA100 with and without S9 mix. Kutlu et al. [13] reported that the presence of mutagens caused frameshift and base-pair substitution mutations in water and sediments of the Porsuk River by Ames test. The previous studies supported that the Ames test can be applied in assessing the mutagenicity of organic extracts from black-odor river.

In this study, a set of combined tests including acute toxicity tests and Ames tests were employed to assess the comprehensive impact of pollutants from Shengtongbang river on aquatic ecological system before and after the remediation. The acute toxicity was performed with *Chlorella vulgaris* and *Daphnia magna*. The mutagenicity assessment of the organic extracts of the water was performed by Ames test. The results are expected to assess the toxicity changes before and after remediation and ecological safety for black-odor river, and further to improve the remediation process based on the biological toxicity results.

## 2. Materials and Methods

### 2.1. Sampling Sites and Remediation Process

The urban black-odor river in this study was located in Shentongbang, Luoyang town, Wujin district, Changzhou city, China. According to the preliminary investigation, there were three natural villages and 64 factories along the Shentongbang river. As a result of the weak environmental awareness among local residents and manufacturers, the direct discharge of domestic and industrial sewage, improper diversion of rain and sewage, and the existence of leakage years ago had caused the deterioration of water quality even if there existed wastewater treatment plants. Although the regulation of sewage discharge has been strengthened and there was no existence of illegal discharge of sewage after remediation, however, contaminants discharged before are still being deposited in the sediments and can be released to the waterbody, causing harm to aquatic life. A lot of black clumps with fetid odor can be seen floating on the river. It was suggested that there might exist organic pollution, while the concentrations of heavy metals (Cr, Cu, Mn, Pb, Zn) detected by inductively coupled plasma–mass spectrometry (ICP-MS) did not exceed Chinese national standard [14]: Environmental Quality Standards for Surface Water (GB3838-2002) for class II.

Shentongbang river was thoroughly applied by several remediation measures. First, through the optimized control of the sluice, a certain exchange of water can be carried out between Wujin Port and Shentongbang river, thereby improving the water quality. Then, the discharge of the factory’s legacy sewage outlets was found out and strictly prohibited, the decentralized domestic sewage was centralized and treated, ecological floating beds and immobilized microorganisms were applied to block flow, settle, and intercept pollutants entering the river. Finally, sediment ecological slope protection technology and planting of aquatic plant at vertical barge vertical were also carried out.

**Figure 1:**
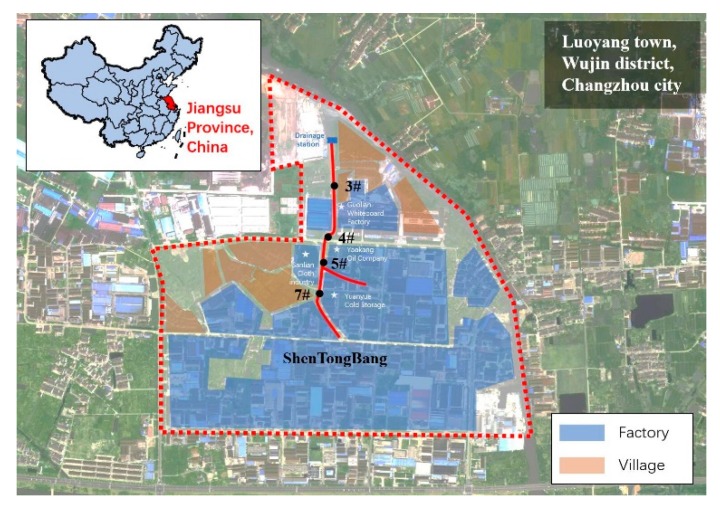
Sampling sites of overlying water in Shentongbang.

Four sampling sites (3#, 4#, 5#, 7#) on Shentongbang were chosen to evaluate the biological toxicity of overlying water before and after remediation (Figure 1). Overlying water is the supernatant in river, which also called covering water [8]. Site 3# (N 31°40′23″, E 120°4′2″) was beside Guolian whiteboard factory with four discharge outlets directly to the river. Site 4# (N 31°40′15″, E 120°4′0″) was under a bridge near Yaokang oil company. There were five discharge outlets along the river. Site 5# (N 31°40′12″, E 120°3′59″) was in Sanlian cloth industry Co., Ltd., where the tributaries flowed into the main stream. Site 7# (N 31°40′8″, E 120°3′59″) was next to Yuanyue cold storage plant where three discharge outlets were along the river.

The water samples were collected over a 24 h period according to standard sampling methods [15]. The environmental parameters on sampling site, such as temperature, pH and dissolved oxygen (DO) were measured. The water samples were sealed, then transported to the laboratory as soon as possible. The airtight water bottles were stored in dark at −20 °C for the toxicity tests.

### 2.2. Physicochemical Analysis of Water Quality

The overlying water was filtered with 0.45 μm filter paper. Chemical oxygen demand (COD), ammoniac nitrogen (NH_4_^+^-N), total nitrogen (TN), and total phosphorus (TP) were analyzed according to standard methods for the examination of water [16].

### 2.3. Toxicity Tests

#### 2.3.1. Acute Toxicity Experiments with *C. vulgaris*

*C. vulgaris* (FACHB-8) was obtained from the Freshwater Algae Culture Collection of the Institute of Hydrobiology, Chinese Academy of Sciences (Wuhan, China). The algae were cultured for three generations in the laboratory before the toxicity experiments. Algal cells in the logarithmic growth phase were used for toxicity experiments, which were conducted in an illumination incubator to maintain the same condition: light: 4000–6000 lx, temperature: 23 ± 2 °C, pH: 7.1.

Ninety-six hours of acute tests by *C. vulgaris* were conducted according to Chinese national standard [17]: Chemicals-Alga Growth Inhibition Test (GB/T21805-2008). The dilution water was used to make a series of concentrations of the overlying water for the experiments. The dilution water had the same hardness and alkalinity as the overlying water, which was prepared according to USEPA 2002. The initial concentration of algae in the solution was 5 10^4^ cells/mL. Three parallel lines were set for each group, including a blank control group (BG11 medium, NC) for analysis. The correlation between algal density and absorbance at 680 nm were plotted before the experiment. During the test period, the absorbance was measured every 24 h, and the density of algae was calculated based on the density-absorbance curve. The test lasted for 96 h.

The specific growth rate μ was calculated as:

(1) $$\mu =\frac{\ln x_{f}-\ln x_{0}}{t_{f}-t_{0}}$$

where *t*_0_ is the beginning time of the test, *t_f_* is the ending time, and *x*_0_ is the density of algae at the beginning, *x_f_* is the density of algae at the ending.

A toxic unit (TU) approach was employed to calculate the mixed toxicity of all toxicants in an effluent, which were calculated based on the formulas below [18]:

(2) $$TU=\frac{100\%}{LC_{50}\left(EC_{50}\right)}$$

where *LC*_50_ or *EC*_50_ is the dilution or concentration of water samples when half of the aquatic organisms are dead or showed growth inhibition.

The acute toxicity results were classified according to the following toxicity classification system [19], as shown in Table 1:

#### 2.3.2. Acute Toxicity Experiments with *D. magna*

*D. magna* was obtained from the Institute of Hydrobiology, Chinese Academy of Sciences. They are cultured in the laboratory, and tested for sensitivity to potassium dichromate before the toxicity experiments. *D. magna* at 6–24 h old were employed in the toxicity test. The experiments were conducted using an illumination incubator to maintain the same conditions: 19 ± 1 °C, DO ≥ 2 mg/L, pH 7.0–8.0, and a 16:8 light: dark cycle with light intensity <1000 lx.

Forty-eight hours of acute tests by *D. magna* were conducted according to Chinese national standards [20]: Method for Acute Toxicity Test of Daphnia Magna Straus (GB/T 16125-2012). Before the experiments, the sensitivity of *D. magna* was tested with potassium dichromate and the results met the quality control requirements for *D. magna* acute toxicity tests. In formal experiments, a series of concentrations of 40-mL experimental solutions (overlying water diluted by percentage with aerated dilution water) were prepared in a 100-mL glass beaker. Four concentration groups were set for each point, including the solvent control group (aerated dilution water, NC). Three parallel lines were set for each group and ten *D. magna* were added to each glass beaker. The numbers of dead *D. magna* were recorded every 24 h, and the mortality rates were calculated. The test was lasted for 48 h.

The mortality rate I was calculated as:

(3) $$I=\frac{N}{N_{0}}\times 100\%$$

where *N*_0_ is the number of *D. magna* at the beginning, *N* is the number of dead *D. magna* after 48 h.

The toxic unit (TU) and classification criteria for acute toxicity (48 h) with *D. magna* was the same as *C. vulgaris*.

If the mortality in a 100% effluent concentration was between 10% and 49%, the TUs were derived as follows:

(4) TU=0.02×mortality(%)

A toxic unit of zero was allocated to mortalities between 0% and 10% in 100% effluent exposure [21].

#### 2.3.3. Ames Test

The mutagenicity of overlying water organics was tested by Ames test. The organic chemicals in the overlying water were extracted by a solid-phase extraction method. Oasis HLB sorbent has been reported with good retention for both polar and non-polar compounds (Liu et al.). This kind of sorbent allow efficient extraction of different pesticides or environmental contaminants such as 2,4-Dichlorophenoxyacetic acid (2,4-D), trichlorophenol (TCP), polycyclic aromatic hydrocarbons (PAHs), herbicides (i.e., atrazine), etc. Waters Oasis HLB cartridges (6 cc, 200 mg sorbents) were conditioned using 10 mL methanol, followed by 10 mL Milli-Q water. Then, 1 L of each water samples was passed through the HLB cartridge at a speed of 3–5 mL/min. After the samples were loaded, 10 mL of Milli-Q water was used to wash each cartridge. The HLB cartridges were then eluted by 10mL hexane/acetone (1:1 by volume), 10 mL dichloromethane and 10 mL methanol in sequence. The three eluates were combined and dried under a gentle nitrogen stream, and the final extract was reconstituted in 1 mL dimethyl sulfoxide (DMSO) for the next mutagenicity experiment.

Four *S. Typhimurium* strains, TA97, TA98, TA100, TA102 were employed in Ames test. The first two strains primarily detect the genotoxic substance of frameshift mutants and the other two strains detect DNA as a mutagenic substance for base pair substitution. The strains were provided by Jiangsu Provincial Center for Disease Control and Prevention and Guizhou Medical University. Rat liver microsomal (S9) was purchased from CHI Scientific, Inc., (Jiangsu, China). Biological characterization, preservation, mutagenesis procedures of these four *S. Typhimurium* strains were with reference to Chinese standards [22]: Safety and Technical Standards for Cosmetics (2015). The positive control experiments were conducted to demonstrate the sensitivity of the strains.

The mutagenicity ratio (MR) was used to determine the genetic toxicity of the water samples, which was calculated as:

(5) $$MR=\frac{X}{X_{0}}$$

where *X* is the number of surviving colonies on organic extract-treated plates and *X*_0_ is the number of the negative control group (NC).

When the mutagenicity ratio (MR) ≥ 2, with the dose-response relationship and the background was normal, the mutagenesis is judged as positive.

### 2.4. Statistical Analysis

Origin Pro 9 software (Origin Lab, Northampton, MA, USA) was applied for data processing. IBM SPSS version 20 software (IBM Corp, Armonk, NY, USA) was applied for the calculation of 96-h EC_50_ and 48-h LC_50_ value with their 95% confidence intervals. The results of Ames tested for overlying water organic were expressed as the Mean ± SD.

Statistical significance was evaluated with one-way analysis of variance (ANOVA) followed by Dunnett’s multiple comparison tests (comparison between NC and water samples, *p* < 0.05 or *p* < 0.01).

## 3. Results and Discussion

### 3.1. Water Quality

The water quality of the overlying water before and after remediation are shown in Table 2. The results indicated that before remediation, COD, TN, TP, NH_4_^+^-N, and DO exceeded the environmental quality standards for surface water (GB3838-2002) seriously. The black-odor river was classified as substandard V water body. After remediation, the COD from all sites was reduced from 49.66–91.80 mg/L to less than 50 mg/L, the TN, TP, and NH_4_^+^-N were also removed effectively after remediation. DO was obviously improved due to the remediation, with all sites meeting the IV even II standard (GB3838-2002) except for site 7#. However, the TN and TP of most sites were still higher than V standard. Only TP from 3# met the index of case-V water. And all parameters of water from 7# were exceeded V standard except pH.

Generally speaking, the water quality was improved significantly by the remediation, but there were still two problems. The first was that the TN and TP from the water were higher than the V standard after the remediation. It was believed that the water eutrophication was serious. The second was that the water from site 7# after the remediation still belonged to substandard V water body, indicated that the pollution caused by the cold storage plant was serious.

### 3.2. Acute Toxicity of Overlying Water before and after Remediation

#### 3.2.1. Acute Toxicity on *C. vulgaris*

Table 3 is the result of 96-h specific growth rate of *C. vulgaris* for overlying water before and after remediation. After 96 h, the specific growth rates of *C. vulgaris* in overlying water from all sites were higher than that of the control group no matter remediation or not. The result indicated that the overlying water promoted the growth of *C. vulgaris*. According to the water quality measurement results, it is speculated that the excessive TN and TP in water provided excessive nutrients for *C. vulgaris*, so the growth rate in overlying waters were higher than the control group. The promotion effect was ranked from large to small respectively: 4#, 3#, 5#, 7#, which was consistent with the water quality results (Table 1). Site 4# showed highest promotion effects, since it was least polluted according to water quality analysis, while site 7# showed lowest promotion because of its worst water quality.

The result indicated there was no inhibitory effect on *C. vulgaris* by overlying water, whatever before or after the remediation. It was believed that the promotion effect by water eutrophication exceeded that of water toxicity on *C. vulgaris*. The results suggested the advantage of biological toxicity tests, which can not only test the overall toxicity of all pollutants contained in waters, but also test the effect of the nutrient elements from waters by the growth rate of algae. The results indicated that the restrictions on the TN and TP are still needed in the future.

#### 3.2.2. Acute Toxicity on *D. magna*

Table 4 is the 48-h LC_50_ on *D. magna* by overlying waters. When the proportion of raw water in all spots was 100%, the mortality rate of *D. magna* did not exceed 50%, which could calculate the TU and evaluate toxicity according to Equations (2) and (4).

According to the toxicity classification system [19], the toxicity of the overlying water to *D. magna* from 3# was slightly toxic before the remediation of the river. The other sites were non-toxic. The comprehensive toxicity of the overlying water to *D. magna* varied from strong to weak from sites 3#, 7#, 5#, and 4#. After the remediation, the overlying water of all sites showed no toxic effect on *D. magna*, especially for site 4#, there was no death of *D. magna* caused by overlying water.

From the results, the TUs of most spots decreased after remediation compared to those before remediation, proved that the toxicity of the overlying water decreased by the remediation measures, especially, the TU of site 4# after remediation was 0, lower than other spots. These toxicity results are all consistent with the improvement of water quality (Table 2), suggesting the remediation on the urban black-odor river is effective. Before the remediation on site 3#, the overlying water had light lethal effect on *D. magna*, it could be related to the contaminants discharged from the whiteboard factory. It is known that the production of whiteboard is inseparable from plastics, and the waste water discharged from the whiteboard factory may contain microplastics and plasticizers that are difficult to degrade. Studies have shown that microplastics can be ingested by zooplankton in the environment and affect the growth and feeding ability of the organism [23].

Moreover, the toxicity result is inconsistent with the results of *C. vulgaris*. From the results above, the TN and TP in the overlying water exceeded the China’s water-quality standard (GB3838-2002). In fact, the effects of these two elements on *C. vulgaris* and *D. magna* were different. In principle, N and P are necessary nutrients for algae *C. vulgaris*, which will promote the growth of *C. vulgaris*, while the excessive TN and TP may have certain toxic effects on *D. magna*. The result not only reflected the changes before and after remediation but also the different effects on different species of aquatic organisms.

### 3.3. Mutagenicity of Overlying Water

#### 3.3.1. Ames Test of Overlying Water Extracts Before and After Remediation

**Figure 2:**
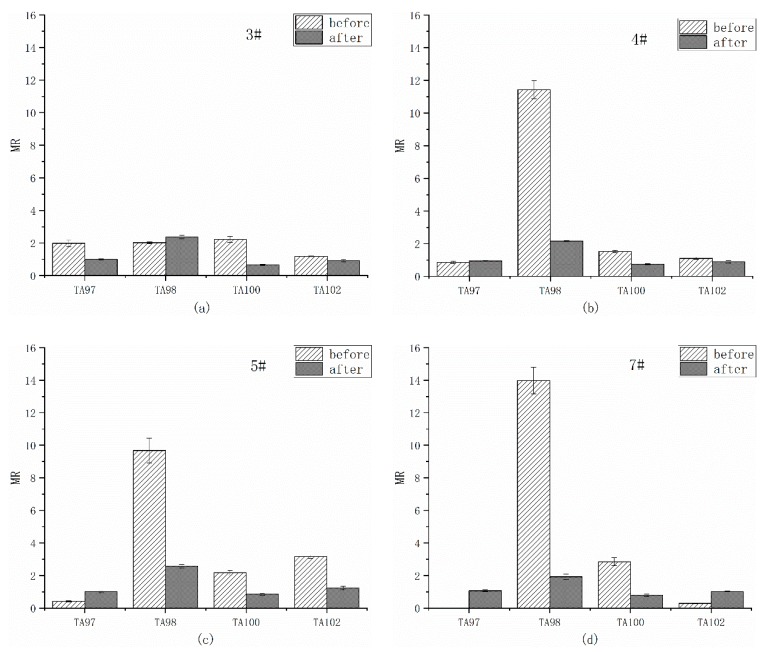
Mutagenesis of four strains by overlying water before and after remediation in the absence of liver microsomal S9; (**a**) site 3#; (**b**) site 4#; (**c**) site 5#; (**d**) site 7#. The mutagenicity ratio (MR) is the average ratio from 3 independent experiments.

Table 5 is the reverse mutation colonies of four *S. Typhimurium* strains detected by organic extracts of overlying water from four sites by Ames tests. The number of reverse mutation colonies in blank and DMSO controls are in the reasonable numbers, which are proposed in the Ames bacterial mutagenicity assay [22], proving that the dissolution of organics by DMSO would not add any mutagenicity to the extraction. The MR values at every site before and after remediation are compared in Figure 2.

On site 3# (in Figure 2a), before remediation, MRs of TA98 and TA100 were slightly higher than 2, indicated the mutagenicity of organic extracts from overlying water. After remediation, the TA98 strain still showed weak positive, while there was no mutagenicity detected by other strains. The MRs of TA97, TA100 and TA102 strains decreased by the remediation, implying the remediation was effective. Notably, for TA98 strain, the MR of after remediation was slightly higher than that of before, showing the possible conversion of organics in water.

As for the toxicity type of site 3#, it may be related to the plasticizers in wastewater from the whiteboard factory. The plasticizers are essential additives in the production of plastic whiteboards. The plasticizers such as phthalate chemicals [24] and bisphenol A [25] have been proved mutagenic by Ames test and other microbial experiments, which may contribute to the mutagenicity of water near whiteboard factory. As for the increase of MRs of TA98 strain, it could result from the release of organics absorbed in sediments because organics with hydrophobic nature and weak degradation characteristics had accumulate in sediments [26]. After the equilibrium between sorbed and aqueous phases was broken, the absorbed organics could repartition into water and cause secondary pollution to the surroundings [27,28].

On site 4# (Figure 2b), before remediation, the MR of TA98 strain was much higher than 2, exhibited a significant mutagenicity, but there was no mutagenicity detected by other strains. After remediation, the TA98 strain still showed weak positive result, but declined obviously (*p* < 0.01). The other strains showed negative results before remediation, and also the MRs of TA100 and TA102 strains dropped a little after remediation. These results all implied that the remediation of black-odor river reduced the mutagenicity of overlying water.

As for the toxicity type of site 4#, this could be resulted from the vegetable oil company. The vegetable oil wastewater belongs to organic wastewater, which contains mainly oil-organic matter, a small amount of protein colloids, pigments, inorganic salts and trace amounts of alkanes, cycloalkane, olefins, and aromatic (PAH) compounds. These organics are difficult to degrade in natural waters in a short time, thus may contribute to the mutagenicity of overlying water.

On site 5# (Figure 2c), before remediation, the MRs of TA98, TA100, and TA102 strains were all higher than 2, showing the mutagenicity from the overlying water to the three strains. For TA97 strain, the number of reverse mutation colonies before remediation was lower than that in the blank group (Table 5). It may be an DNA or cell level damage which inhibited the growth of the strain. After remediation, the number of reverse mutation colonies of TA97 strain returned to normal, and the mutagenicity results still showed negative. Although the mutagenicity on TA98 strain still showed weak positive after remediation, the MR of TA98 decreased significantly (*p* < 0.01), and the MRs of TA100 and TA102 strains also decreased, suggesting the decline of mutagenicity of overlying water by the remediation.

The results from site 5# suggested the complexity of organics in the overlying water. Site 5# is near a cloth factory, the wastewater of the factory is complex and diverse, which cannot be completely removed by the traditional sewage treatment process. The wastewater generally contains additives, detergents, surfactants, and dyes, which are carcinogenic, teratogenic, and mutagenic [29]. These could also contribute to the diversity of mutagenicity results on site 5#.

On site 7# (Figure 2d) before remediation, the overlying water showed strong mutagenicity to strains TA98 and weak mutagenicity to strains TA100, the mutation types were similar to site 5#. After remediation, the MRs of four strains were all less than 2, indicating that the mutagenicity of overlying water at site 7# disappeared by remediation. The TA97 and TA102 strains were inhibited before remediation.

The mutagenicity results on site 7# before remediation may resulted from the organic wastewater from the cold storage plant, since the main organic pollutants in the cold storage plant were petroleum, benzene, etc., and these organic substances all had certain genotoxicity [30,31]. It was speculated that the cold storage plant may discharge these organics into the river before remediation.

From the results, the organics could be possible mutagenicity contaminants at these sites in addition to TN and TP. The Oasis HLB sorbent has been reported with efficient extraction of different pesticides or environmental contaminants, and its recoveries and detection limits in surface waters were 80%–108% and 1–15 ng/L respectively by HLB solid-phase extraction and gas-chromatography/mass spectrometry [32]. It is believed that most of the organics can be extracted from the black-odor river.

In general, positive mutagenicity results were detected from the overlying water of all the sites on TA98, TA100, and TA102 strains before remediation, indicated the diverse mutagenicity types, including the frameshift mutants and base pair substitution. Except the weak mutagenicity of TA98 strain, no mutation was detected from all sites on other strains after remediation. The mutagenicity of organics from the overlying water decreased after remediation, which is consistent with the improvement of water quality and the acute toxicity results of *D. magna*.

Although the acute toxicity of overlying water by *D. magna* showed no toxicity of overlying water of site 4, 5, 7 before remediation, however the Ames test indicated the mutagenicity from the organic extracts from these sites, it is proved that there is still an genetic toxicity risk existing in the waterbodies. So the mutagenicity assays can be considered as an effective tools for environmental monitoring and choosing appropriate treatment processes, such as active carbon or advanced oxidation processes [33]. In other words, the ecological restoration of black-odor river still needs to be improved to eliminate this kind of mutagenicity.

#### 3.3.2. Ames Test of Overlying Water after Remediation in the Presence and Absence of Liver Microsomal S9

To verify whether the toxicity of mutagenic contaminants in the overlying water will be changed by the metabolism of the microorganisms, the liver microsomal S9 were introduced to the Ames tests, for those water samples with negative mutations after remediation.

**Figure 3:**
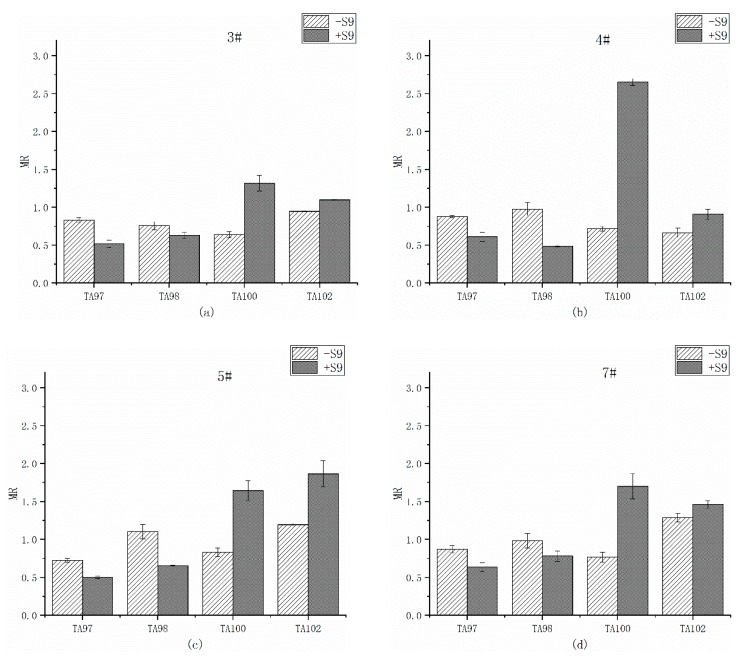
Mutagenesis of four strains by overlying water in the presence and absence of liver microsomal S9; (**a**) site 3#; (**b**) site 4#; (**c**) site 5#; (**d**) site 7#. The mutagenicity ratio (MR) is the average ratio from 3 independent experiments.

Table 6 is the reverse mutation colonies of four *S. Typhimurium* strains detected by overlying water organic extracts after remediation. The MR values in the presence (+S9) and absence (–S9) of S9 at four sites are compared in Figure 3. For TA97 strain, the mutation colonies in DMSO were a little higher than that of blank control. However, there was no significant mutagenicity detected from DMSO group, proved that the dissolution of organics by DMSO would not add any mutagenicity to the extraction.

On site 3# (Figure 3a), the MR values of four strains were all less than 2, both with and without S9, indicating that the overlying water after remediation had no mutagenicity to four strains. Additionally, the MRs of TA100 and TA102 strain raised slightly when added S9.

On site 4# (Figure 3b), in the presence of S9, the MRs of TA100 and TA102 strain raised. In particular, the MR of TA100 strain was over 2 and 3 times compared to that in the absence of S9. The result indicated that the mutagenicity of organics was increased after metabolism.

For site 5# and 7# (Figure 3c,d, respectively), the results were similar to that on site 3#. There was no mutagenicity detected for four *S. Typhimurium* strains both in the presence and absence of S9. And the change of MRs of four strains was also as same as that on site 3#.

From the results above, the addition of S9 metabolized organics in overlying water, increasing the mutagenicity (site 4# to TA100). The fact revealed that the organic chemicals present in the overlying water will be changed by the metabolism of microorganism and therefore cause potential risk to aquatic ecosystem. Furthermore, the process of the bio-concentration, bio-accumulation, and bio-amplification in the natural ecosystem may also be a threat to the aquatic life. Thus, there is a need for more stringent monitoring of such ecological risk to predict or prevent the damage to the ecosystem in the future.

In acute toxicity tests by *C. vulgaris* and *D. magna* and Ames tests by four *S. Typhimurium* strains, the different level toxic tests were employed in the present study to reveal the different effects of toxicants from the overlying water.

In conclusion, the overlying water was not highly toxic to *C. vulgaris* and *D. magna,* but had certain mutagenicity to four *S. typhimurium* strains, especially for the TA98 and TA100 strains which could cause frameshift and base-pair substitution mutations. Although the mutagenicity was detected from the organic extract of the overlying water, it confirmed that there did exist some mutagenic contaminants in the overlying water, even after remediation. It could be predicted that the contaminants with no mutagenicity in the environment could be changed by the microbial metabolism. So, the assessment of remediation effect for black-odor river should be monitored in a long time to observe the possible ecological risk. The comprehensive assessment of the impact of the black-odor river through multiple levels toxicity tests would provide a scientific method for assessing the safety of natural ecosystem in the future.

## 4. Conclusions

- Acute toxicity of overlying water from Shengtongbang by *C. vulgaris* tests showed nontoxic before and after remediation, but promoted the growth of *C. vulgaris* because of the excessive TN and TP, indicated that the restrictions on the TN and TP are still needed.
- The *D. magna* tests showed the toxicity of overlying water on site 3# before remediation, and be declined to nontoxic after remediation. The results suggested the remediation effects were effective for *D. magna.*
- The mutagenicity of organic extracts from overlying water by Ames test at all sampling sites was positive before remediation. After remediation, the mutagenicity of most sites eliminated except of the TA98 strain from 3 of 4 sites. The addition of liver microsomal S9 induced the positive mutagenicity on site 4#, implying that the metabolism of the microorganisms changed the mutagenicity of the organic extracts from overlying water. The remediation significantly improved the water quality, but the potential ecological risk still exists in Shengtongbang.
- The results of this study applied comprehensive toxicity tests to evaluate the remediation results in urban black-odor rivers, and provided biological assessing method for the remediation evaluation.

## Figures and Tables

**Table 1:** Toxicity classification system.

TU	Classification	Toxicity
TU < 0.4	I	no acute toxicity (NT)
0.4 ≤ TU < 1	II	slight acute toxicity (ST)
1 ≤ TU < 10	III	acute toxicity (AT)
10 ≤ TU < 100	IV	high acute toxicity (HT)
TU ≥ 100	V	very high acute toxicity (VT)

**Table 2:** Physicochemical parameters of the overlying water before and after remediation.

Parameters (mg/L)		COD	TN	TP	$NH_{4}^{+}\text{-}N$	DO	pH (Non-Dimensional)
Before remediation	3#	55.68 *	13.05 *	0.723 *	5.007 *	0.58	7.32
	4#	54.18 *	13.97 *	0.832 *	6.085 *	0.49	7.51
	5#	49.66 *	11.51 *	0.604 *	7.533 *	0.63	7.46
	7#	91.80 *	15.85 *	1.624 *	9.694 *	0.77	7.29
After remediation	3#	34.55	9.55 *	0.355	1.908	6.970	7.71
	4#	28.73	10.15 *	0.406 *	1.426	3.570	7.94
	5#	36.15	9.89 *	0.447 *	1.828	4.940	8.12
	7#	46.64 *	14.41 *	1.071 *	2.015 *	1.64 *	8.17

* Exceed the China’s water-quality standard for class V (GB3838-2002).

**Table 3:** Specific growth rate (96-h) of *C. vulgaris* to overlying water before and after remediation.

Time	Before Remediation	After Remediation
Sites	Specific Growth Rate (96 h)	
3#	89.11% ± 6.95% *	91.04% ± 4.44%
4#	93.80% ± 1.34% **	94.01% ± 2.36% *
5#	88.55% ± 2.69%	92.62% ± 3.71% *
7#	86.32% ± 0.73%	88.47% ± 5.82%
NC	81.47% ± 1.85%	85.27% ± 1.44%

Comparison between NC (BG11) and water samples: *. *p* < 0.05, ** *p* < 0.01.

**Table 4:** Biological toxicity assessment on overlying water by *D. magna* before and after remediation.

Sites	3#		4#		5#		7#	
	Before	After	Before	After	Before	After	Before	After
48-h LC_50_^a^	218.67%	299.94%	629.80%	/	519.70%	500%	311.64%	1515.15%
TU	0.46	0.33	0.16	0	0.19	0.20	0.32	0.07
Toxicity	ST	NT	NT	NT	NT	NT	NT	NT

^a^ The 48-h LC_50_ of *D. magna* was estimated by concentration proportion of raw water; There was no dead *D. magna* caused by raw water; ST = slight acute toxicity, NT = no acute toxicity.

**Table 5:** Reverse mutation colonies of four strains detected by overlying water extracts before and after remediation.

Samples	TA97		TA98		TA100		TA102	
	Before	After	Before	After	Before	After	Before	After
3#	373 ± 31.5	189 ± 7.0	88 ± 2.8 *	104 ± 4.9 *	419 ± 35.4 *	125 ± 7.8	365 ± 6.4	287 ± 23.4
4#	160 ± 15.7	165 ± 4.9	497 ± 24.0 *	94 ± 1.4 *	286 ± 14.1	139 ± 5.7	340 ± 20.7	277 ± 23.3
5#	79 ± 5.7 ^a^	190 ± 11.1	421 ± 33.1 *	112 ± 4.6 *	412 ± 26.9 *	162 ± 11.0	979 ± 31.1 *	380 ± 33.9
7#	0 ^a^	201 ± 11.3	608 ± 35.4 *	84 ± 7.0	538 ± 45.2 *	149 ± 12.7	90 ± 6.8 ^a^	316 ± 8.5
Blank	185 ± 10.6		45 ± 2.1		181 ± 4.4		328 ± 10.6	
DMSO	191 ± 10.8		43 ± 3.5		196 ± 11.1		291 ± 1.4	

^a^ bacterial inhibition effect; * MR ≥ 2 compared to control.

**Table 6:** Reverse mutation colonies of four strains detected by overlying water after remediation in the presence and absence of liver microsomal S9.

Samples	TA97		TA98		TA100		TA102	
	−S9	+S9	−S9	+S9	−S9	+S9	−S9	+S9
3#	255 ± 9.2	159 ± 14.7	51 ± 3.5	42 ± 2.8	125 ± 7.8	256 ± 20.2	323 ± 1.4	375 ± 1.4
4#	269 ± 4.9	187 ± 18	65 ± 5.7	31 ± 2.1	139 ± 5.7	517 ± 9.2 *	227 ± 21.2	310 ± 22.6
5#	222 ± 7.1	164 ± 16.3	74 ± 6.4	44 ± 0.7	162 ± 11.0	320 ± 25.5	408 ± 5.7	638 ± 59.4
7#	267 ± 14.1	196 ± 17.7	66 ± 6.4	52 ± 4.6	149 ± 12.7	331 ± 32.5	440 ± 19.2	500 ± 17.7
Blank	182 ± 12	166 ± 11.9	53 ± 4.9	51 ± 1.4	181 ± 4.4	205 ± 6	321 ± 11.3	342 ± 1.4
DMSO	305 ± 10.6	309 ± 19.1	62 ± 2.8	72 ± 3.5	196 ± 11.1	194 ± 9.1	340 ± 26.9	344 ± 11.3

* MR ≥ 2 compared to control.
